# Unveiling the Mechanism: Injectable Poly‐L‐Lactic Acid's Evolving Role—Insights From Recent Studies

**DOI:** 10.1111/jocd.16635

**Published:** 2024-10-16

**Authors:** Luiz Eduardo Avelar, Schafiq Nabhani, Stas Wüst

**Affiliations:** ^1^ Private practice Belo Horizonte Brazil; ^2^ Police Department of Minas Gerais State Belo Horizonte Brazil; ^3^ Clinic Domani Belo Horizonte Brazil; ^4^ Galderma Laboratorium GmbH Düsseldorf Germany; ^5^ Z282 Medical Affairs Consulting Beverly Massachusetts USA

For over 25 years, Sculptra poly‐L‐lactic acid—(PLLA‐SCA; Galderma Sweden) is used in aesthetic dermatology, addressing volume loss, skin laxity, and wrinkles effectively [1]. This synthetic, bio‐compatible product is available as a lyophilized powder with PLLA‐SCA microparticles, mannitol, and sodium carboxymethylcellulose, and is prepared as a suspension using sterile water for injection [2].

Biocompatible, polymeric biomaterials like PLLA, when implanted, induce a foreign body reaction shaped by the material's characteristics, patient specifics, and implantation technique. Controlling these factors ensures a predictable host response, crucial for achieving desired outcomes in collagen stimulation treatments.

While the specific molecular mechanisms of PLLA's stimulation of collagen production are not fully understood, this article reviews literature on PLLA‐induced biochemical pathways in fibroblasts, adipocytes, and macrophages, exploring their interplay. Variations among injectable PLLA formulations, particularly in tissue integration and degradation rates, are noteworthy [3, 4]. This article differentiates between PLLA‐SCA and other PLLA formulation.

Fibroblasts, known for their involvement in the assembly and maintenance of the extracellular matrix (ECM), have been recognized as key players in the PLLA mechanism of action. In 2012 Courderot‐Masuyer et al. [5] presented the first evidence that the addition of 0.1% PLLA‐SCA to the growth medium of ex vivo human fibroblasts, derived from female wrinkles, leads to an increase in collagen Type I production. Although this initial study is limited by the sample size of three donors, its findings have been supported by subsequent research.

Goldberg et al. [6] substantiated the idea of PLLA‐SCA‐induced collagen neogenesis in humans. In their clinical study involving 14 subjects, they demonstrated that the subdermal application of PLLA‐SCA significantly enhances collagen Type I production. Notably, collagen Type I levels increased by 65.5% after 3 months, with a slight decrease at the 6‐month mark, illustrating a sustained effect.

Kim et al. [7] explored whether PLLA‐SCA could trigger collagen synthesis in fibroblasts.

Cultured human fibroblasts (Cell line Hs68) showed a significant increase in collagen Type I RNA and procollagen in cell extracts, and collagen Type I in the medium after a 48‐h exposure to 0.1% PLLA‐SCA. Two independent studies substantiated these results. While Huth et al. [8] reported a significant thickening of their skin model after PLLA‐SCA‐application, Zhu et al. [9] confirmed that increasing concentrations of PLLA‐SCA in the growth medium (0.1%, 0.5%, and 1%) resulted in a gradual increase in COL1A1 (collagen 1 alpha 1) and COL1A2 mRNA, as well as COL1A1 protein in the same human cell line.

These studies also highlighted PLLA‐SCA's broader anabolic effects on the ECM, evidenced by similar results for elastin—a crucial crosslinking protein between collagen fibers—and changes in TIMP‐1 and ‐2 (tissue inhibitor of metalloprotease) mRNA levels and a dose‐dependent reduction in MMP‐1 (matrix metalloprotease 1) mRNA levels.

These effects have been linked to the TGF‐β (transforming growth factor beta) signaling pathway. Increased levels of TGF‐β1, phosphorylated SMAD‐1 and ‐2 proteins were detected after stimulation with 0.5% PLLA‐SCA. These findings align with previous research, indicating that TGF‐β1 auto‐ and paracrine stimuli regulate various fibroblast functions, including proliferation, migration, connective tissue synthesis, and wound healing, as documented by Ashcroft, Yao, Lee et al. [10, 11, 12]. Huth et al. [13] provided an additional link between TGF‐β signaling and PLLA‐SCA in 3D human skin models.

The current mechanistic observation posits that following the administration of the PLLA‐SCA suspension, immediate volumization is observed, persisting for several days until the carrier solution is fully absorbed, as detailed in Moyle's 2004 research [14]. The remaining PLLA‐SCA particles incite a subclinical inflammatory and foreign body reaction, as described in Junge's 2012 study [15]. This foreign body reaction entails the recruitment of monocytes and their differentiation into macrophages, which coalesce into giant cells, encapsulating the foreign bodies composed of PLLA‐SCA as demonstrated by Goldberg et al. and later by Mazzuco et al. [6, 16]. However, alongside the multinucleated histiocytes surrounding the PLLA‐SCA particles, Goldberg's team also documented the presence of lymphocytes around superficial dermal vessels in some tissue samples. This was characterized as a mild inflammatory reaction, even 6 months post‐injection of PLLA‐SCA in a significant proportion of patients (10 out of 14). The researchers noted that the stimulation of dermal collagen occurred without a notable inflammatory response, suggesting a more complex mechanism. In 2023, Oh et al. [17] provided the foundation for an elegant hypothesis for the coexistence of leukocytes and the absence of an overt inflammatory reaction, attributing it to the polarization of macrophages. Macrophages are known to exist in two subtypes: pro‐inflammatory M1 and anti‐inflammatory, tissue‐regenerating M2 macrophages [18, 19]. M1 macrophages are activated by tissue factors like bacterial lipopolysaccharide and interferon gamma and secrete proinflammatory cytokines (e.g., IL‐1, IL‐6, IL‐12, and IL‐23). In contrast, M2 macrophages, activated by IL‐4 and IL‐13, promote anti‐inflammatory responses and tissue regeneration, secreting factors like TGF‐β.

Using murine models, cultured murine macrophages (RAW264.7), human fibroblasts (CCD‐986sk), and their own PLLA formulation, Oh et al. [17] observed an increase in IL‐4 and IL‐13 post‐PLLA treatment (10 mg/mL), and a shift toward M2 macrophage polarization both in vivo and in vitro. Although, the compared studies differ in their time points and models (Oh: 48 h for cell culture and 28d for mice vs. Goldberg: 3, 6, and 12 months in humans) one possible conclusion is that the leukocytes observed in Goldberg's study were likely M2 polarized macrophages, correlating with the absence of inflammation. Huth et al. could substantiate this result by demonstrating CD‐163^+^ (cluster of differentiation‐163, a marker for M2 polarization) macrophages in their 3D skin models after stimulation with PLLA‐SCA [13]. Furthermore, Oh et al. [17] replicated previous findings, demonstrating an upregulation of TGF‐β, COL1A1, TIMP1, and SMAD2 phosphorylation, alongside a downregulation of MMP2 and MMP3 in cultured fibroblasts and skin biopsies. They also observed PLLA‐induced phosphorylation of AKT in senescent fibroblasts and aged mouse skin, echoing the results of Kim et al. with PLLA‐SCA [7]. As a side note, already activated M2 polarized human macrophage cultures display an increase of two pro‐inflammatory factors (MIP1a and 1b) as a result of incubation with PLLA‐SCA for 24 h [20]. As no other inflammation markers were significantly regulated, this finding likely reflects a short‐term stress response.

Finally, adipocytes, although less understood, also play a role in the context of PLLA‐based treatments. Focusing on PLLA scaffolds for post‐mastectomy aesthetic treatments Ogino et al. [21, 22, 23] have shown a consistent increase in fat mass across different animal models including rats, rabbits, and pigs. They observed increased adipocyte levels at 12 and 24 months in rabbits with PLLA porous capsule implants under inguinal fat, without added growth factors and no growth after 12 months [22]. After 12‐ and 24‐month macrophage invasion was observed in the capsule implants. Using a collagen sponge surrounded by a PLLA‐mesh, the authors reported a very low number of macrophages while demonstrating even stronger effects than with the capsule implant alone. In a porcine model, similar results were achieved: decreased implant size and increased adipose tissue, especially between 6 and 9 months, then reducing after 12 months [23]. The tissue contained adipocytes, collagen fibers, and notably, capillary formation around PLLA threads at 12 months, suggesting PLLA's direct role in this process. Recently Jin et al. described the PLLA induced browning of primary murine fibroblast, but no adipogenic differentiation was observed in mesenchymal stem cells after PLLA stimulation [24]. The authors showed that PLLA increases lactate levels in the culture medium and its uptake via the lactate transporter Mct1/4 is responsible for the browning. Contradictory to this study, Kim et al. demonstrated an effect of injectable PLLA‐SCA on adipogenesis using murine pre‐adipocytes (3T3‐L1 cells) post UVB‐irradiation, simulating deep skin photoaging [25]. They observed significant adipogenesis by Day 7 post‐differentiation, with PLLA‐SCA enhancing collagen Types IV and VIɑ1 production, even without UVB damage. This is especially interesting as collagen Type VIɑ1 has been shown to promote adipogenesis [26] (as well as collagen Type I [27] which is produced by fibroblasts upon PLLA‐SCA‐stimulation). The differences in the studies are likely due to the chosen in vitro models.

In summary, recent studies have advanced our understanding of PLLA's biological effects. Goldberg et al. showed that PLLA‐SCA particles are encapsulated by multinucleated giant cells, and that the presence of PLLA‐SCA leads fibroblasts to secrete TGF‐β, triggering their own activation and proliferation, thus promoting new ECM formation.

Macrophages play a vital role, likely undergoing M2 polarization with PLLA‐SCA, reducing inflammation, and releasing auto‐ and paracrine signals like IL‐4 and IL‐13. This stimulates fibroblast activity via TGF‐β. Additionally, PLLA triggers adipogenesis, which may explain the volume increase in treated areas [28]. PLLA‐SCA prompts collagen VI production in pre‐adipocytes differentiation and Collagen I in fibroblasts, creating a feedback loop for adipogenesis regulation. Finally, there are hints of TGF‐β signaling positively affecting adipocyte differentiation [29] Figure 1.

**FIGURE 1:**
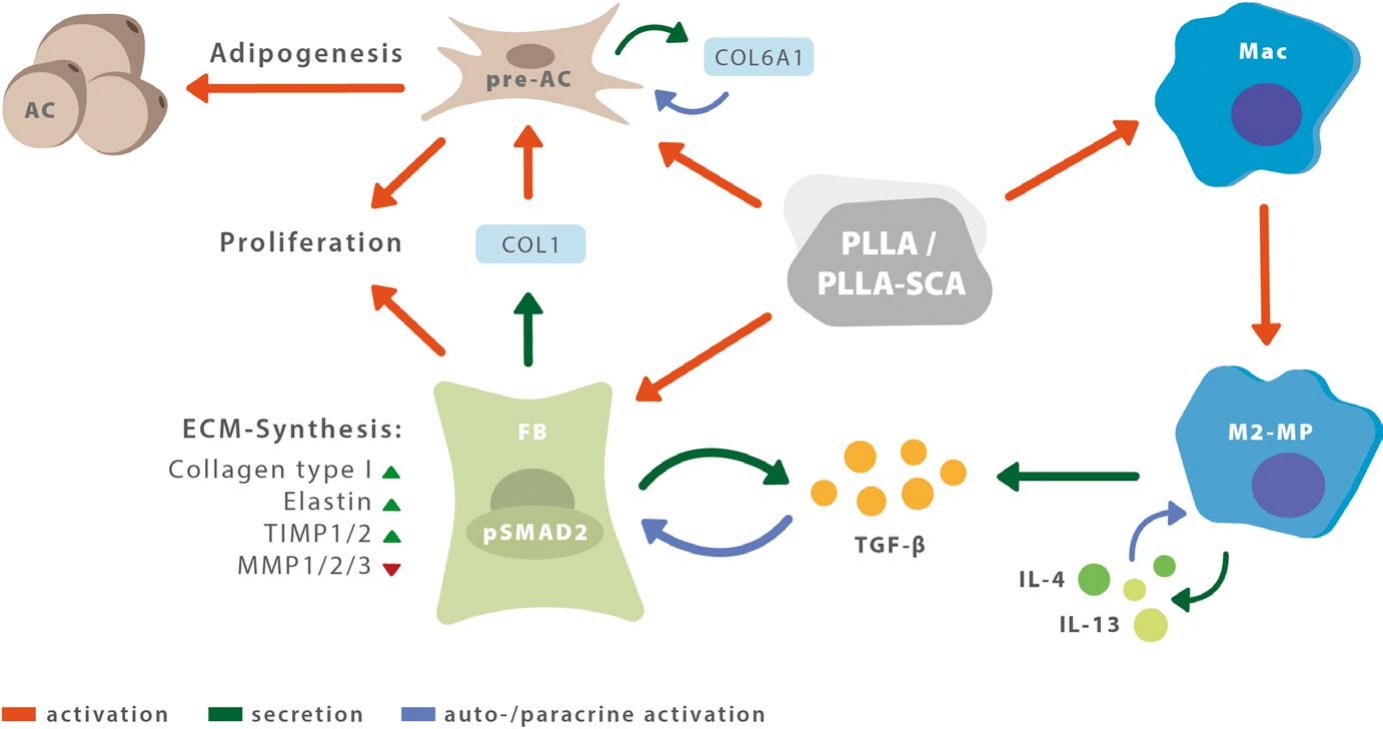
Suggested mode of action of PLLA/PLLA‐SCA (poly‐l‐lactic acid). AC, adipocytes; COL, collagen; ECM, extracellular matrix; FB, fibroblast; IL, interleukin; M2‐MP, M2‐polarized macrophage; Mac, macrophages; MMP, matrix metalloprotease; pre‐AC, preadipocytes; pSMAD, phosphorylated mothers against decapentaplegic homolog; TGF‐β, transforming growth factor beta; TIMP, TIMP metallopeptidase inhibitor.

Despite progress, key questions persist. The precise mechanism of cell recognition of PLLA remains unclear. Further research is needed on PLLA's effects on adipogenesis and on the immunological responses over time and tissue, its direct effect on angiogenesis, and the interaction between multinucleated histiocytes around PLLA particles and other ECM cells.

## Conflicts of Interest

Luiz Eduardo Avelar is an investigator, a speaker, and consultant for Galderma. Schafiq Nabhani is an employee of Galderma. Stas Wüst is a former employee and consultant for Galderma. The submission fees and the submission process were funded by Galderma.

## Data Availability

The data that support the findings of this study are openly available in PubMed (National Library of Medicine, NLM) at https://pubmed.ncbi.nlm.nih.gov/.
